# Impact of Dietary Alginate Hydrogel Capsules with Lavender Essential Oil on Oxidative Stability, Fatty Acid Profile, and Mineral Composition of Breast Muscles in Broiler Chickens

**DOI:** 10.3390/foods14193409

**Published:** 2025-10-02

**Authors:** Michalina Adaszyńska-Skwirzyńska, Paweł Konieczka, Krzysztof Kozłowski, Dorota Witkowska, Yu-Hsiang Yu, Marcin Barszcz, Adrianna Konopka, Mateusz Bucław, Artur Bartkowiak

**Affiliations:** 1 Department of Monogastric Animal Sciences, Faculty of Biotechnology and Animal Husbandry, West Pomeranian University of Technology in Szczecin, Janickiego St. 29, 71-270 Szczecin, Poland; mateusz.buclaw@zut.edu.pl; 2 Department of Animal Nutrition, The Kielanowski Institute of Animal Physiology and Nutrition, Polish Academy of Sciences, Instytucka 3, 05-110 Jabłonna, Poland; pawel.konieczka@uwm.edu.pl (P.K.); m.barszcz@ifzz.pl (M.B.); a.konopka@ifzz.pl (A.K.); 3 Department of Poultry Science and Apiculture, Faculty of Animal Bioengineering, University of Warmia and Mazury in Olsztyn, Oczapowskiego St. 5, 10-719 Olsztyn, Poland; kristof@uwm.edu.pl; 4 Department of Animal Welfare and Research, Faculty of Animal Bioengineering, University of Warmia and Mazury in Olsztyn, Oczapowskiego St. 5, 10-719 Olsztyn, Poland; dorota.witkowska@uwm.edu.pl; 5 Department of Biotechnology and Animal Science, National Ilan University, No. 1, Sec. 1, Shennong Rd., Yilan City 26047, Taiwan; yuyh@niu.edu.tw; 6 Center of Bioimmobilisation and Innovative Packaging Materials, Faculty of Food Sciences and Fisheries, West Pomeranian University of Technology in Szczecin, Janickiego St. 35, 71-270 Szczecin, Poland; artur-bartkowiak@zut.edu.pl

**Keywords:** alginate hydrogel, antioxidant activity, broiler chickens, lavender essential oil, meat, oxidative stability

## Abstract

Poultry meat, due to its high content of polyunsaturated fatty acids, is particularly susceptible to lipid oxidation, which affects its quality and shelf life. Optimizing meat composition, including fatty acid profile and antioxidant activity, is essential for consumer health. The study aimed to evaluate the effect of supplementing hydrogel capsules containing immobilized lavender essential oil (HE group) and capsules without immobilized essential oil (H group) on the oxidative stability, fatty acid profile, and mineral composition of broiler chicken breast muscles. The study results showed that supplementation with the lavender oil hydrogel (HE) significantly reduced total superoxide dismutase (SOD) and CuZn-SOD activity in breast muscles. Although TBARS values did not show significant differences, the reduced SOD activity may indicate decreased free radical production or more effective action of other antioxidant mechanisms. The fatty acid profile was significantly altered, with a lower content of saturated fatty acids (SFAs) observed in the HE group. Significant changes were also observed in the mineral composition of the muscles. The HE group had a higher sodium content and lower copper and iron levels compared to the control group. These changes may indicate an effect of the hydrogel and essential oil on mineral metabolism and bioavailability. The study suggests that hydrogels with immobilized lavender essential oil (LEO) may positively affect poultry meat quality by improving its fatty acid profile and oxidative stability, although these mechanisms require further research and confirmation.

## 1. Introduction

In recent years, improving animal welfare and food safety has become a priority in poultry production [1]. The introduction of a ban on the use of antibiotic feed additives as growth promoters in EU countries in 2006 led to the search for alternative substances, including biologically active plant-derived compounds known as phytobiotics [2,3,4,5]. Recent studies indicate that herbs and their biologically active compounds, including essential oils (EOs), may be a promising alternative to antibiotic growth promoters and lipid-protecting agents against oxidative damage [6]. The modern world faces challenges in finding innovative, sustainable, and health-promoting solutions in various economic sectors. To meet these challenges, researchers and industries are increasingly adopting nature-inspired solutions through advanced biotechnology and green chemistry. One promising research direction is the use of alginate hydrogels with immobilized EOs. Hydrogels, with their unique structure that allows high water absorption, are an effective medium for delivering active substances such as EOs [5,6].

Certain physicochemical properties of EOs, such as volatility, sensitivity to light, heat, oxygen, and moisture, as well as low stability and susceptibility to degradation during processing, limit their direct application in many fields. Immobilizing essential oils in hydrogels provides an innovative solution to these challenges [6]. This process involves encapsulating essential oil molecules within a hydrogel matrix, protecting them from external factors and enabling controlled release. Algininate hydrogels, derived from natural polysaccharides such as seaweed, are particularly promising in this context [5]. Alginates are biocompatible, biodegradable, and non-toxic, making them safe for use in the food, pharmaceutical, feed, and agricultural industries [6]. Encapsulating EOs within hydrogel matrices offers multiple functional benefits. A key advantage is the increased stability of bioactive compounds, as the hydrogel matrix protects EOs from oxidation, thermal degradation, and evaporation, thereby extending their shelf life and effectiveness. Another advantage is controlled release of the active substance, as the hydrogel structure allows gradual and regulated delivery of active compounds, which is crucial for optimizing their effectiveness, for example, in drug delivery systems or phytobiotic feed additives. In recent years, interest in the use of phytobiotics has increased due to extensive research evaluating their antibacterial and antioxidant properties in vitro [7,8,9,10] and in vivo, including animal models [6,11,12,13,14]. Lavender (*Lavandula angustifolia*) is an aromatic plant in the *Lamiaceae* family and is an excellent example of a plant with phytobiotic properties that contains numerous biologically active compounds with a broad spectrum of activity [6,15,16]. The available literature also contains information about the antioxidant properties of this plant and its secondary metabolites [7,17,18]. However, there are few studies in the literature demonstrating antioxidant activity under in vivo conditions [13,14]. Antioxidant activity plays a key role in preventing oxidative stress induced by excessive intracellular accumulation of reactive free radicals, such as reactive oxygen species (ROS) and reactive nitrogen species (RNS) [19]. The two free radicals (ROS and RNS) are an integral part of normal cellular function and the host’s defense system against invading bacteria, but their excessive levels contribute to the onset and progression of various pathologies involved in the development of many diseases [19]. Oxidative stress, resulting from an imbalance between free radical production and the body’s ability to neutralize them, is considered a contributing factor in the development of numerous lifestyle-related diseases, including cardiovascular diseases, cancer, and neurodegenerative disorders [20,21].

Poultry meat, particularly of broiler chickens, is an important component of the human diet worldwide due to its high nutritional value, low fat content, and relatively low production costs [22,23,24]. However, broilers, known for their rapid growth and efficient feed conversion, have become more demanding in terms of housing and nutrition [25,26]. Long-term selection for meat poultry has weakened their adaptive mechanisms, making them less resilient to environmental factors and more susceptible to stress, which negatively affects productivity and health [1,24]. Stressful conditions predispose poultry species to increased production of free radicals, while the activity of antioxidant enzymes and the ability to eliminate free radicals decline [27,28,29]. Livestock animals, including poultry, are frequently exposed to stressful conditions such as heat stress, poor housing conditions, harmful substances in feed, and infections, which increase ROS production and oxidative damage. Prolonged exposure of poultry to stressful conditions disrupts the redox balance, leading to qualitative changes in animal-derived products, including meat [30]. Poultry meat is highly susceptible to peroxidation due to its high content of easily oxidizable polyunsaturated fatty acids (PUFAs) in phospholipid structures [31]. During lipid oxidation, secondary products such as peroxides, aldehydes, ketones, and hydrocarbons are formed, which cause the sharp, rancid odor and flavor of meat, also frequently affecting its organoleptic properties [32].

Proteins are also susceptible to oxidative modifications induced by reactive oxygen species (ROS). Protein oxidation causes changes in hydrophobicity, conformation, solubility, and susceptibility to proteolytic enzymes, resulting in reduced digestibility. Protein oxidation also reduces the absorption of amino acids essential to humans, thereby diminishing the nutritional value of meat [33]. Moreover, oxidative changes in protein structure reduce the water-holding capacity of meat, complicating its processing. Therefore, in the context of growing demand for healthy food, natural feed additives for animals are gaining increasing importance as they help neutralize free radicals and reduce oxidative stress. The antioxidant properties of animal feed additives are crucial for meat quality and shelf life, as well as their potential impact on consumer health [28,34,35]. Therefore, the search for natural and effective antioxidant strategies is highly important. The aim of the study was to evaluate the effect of dietary supplementation with alginate hydrogel capsules containing immobilized LEO on antioxidant enzyme activity, fatty acid profile, and mineral content in the breast muscles of broiler chickens.

## 2. Materials and Methods

### 2.1. Essential Oil and Alginate Hydrogels Capsules

Lavender (*Lavandula angustifolia*) essential oil was obtained from a commercial supplier (Avicenna Oil, Wrocław, Poland). To characterize its chemical profile, the sample was subjected to gas chromatography–mass spectrometry (GC-MS) analysis using an Agilent apparatus (6890N/5973N, Santa Clara, CA, USA) coupled with a 5973 Network mass detector and a 7683 Series Injector autosampler. Separation was carried out on an HP-5MSI capillary column (30 m × 0.25 mm). The injector was maintained at 250 °C, the ion source at 230 °C, and the quadrupole at 150 °C. The analysis was conducted with a 3 µL sample injection in a 10:1 split mode. The GC oven program began at 60 °C for 3 min and was subsequently increased by 10 °C per minute to a final temperature of 300 °C and held for 13 min. The mass spectrometer was operated in full scan mode (mass range: *m*/*z* 40–500) with an ionization energy of 70 eV. The individual components were identified by cross-referencing their mass spectra with a standard database (NIST 04), requiring a minimum similarity match of 95%. Further confirmation was achieved by calculating and comparing their retention indices against those of a C7-C30 *n*-alkane standard series [36]. The relative abundance of each compound, expressed as a percentage, was computed based on the integrated peak areas from the total ion chromatogram, with results representing the mean of three analytical replicates. For subsequent application, the lavender oil (0.4 mL/L) was first emulsified with an equal volume of Tween 80 and then integrated into alginate hydrogel capsules obtained from APRS S.A. (Nielbark, Poland).

### 2.2. Bird Trial

The study was carried out on a commercial broiler production facility (Veterinary Identification No: 32044946; Żabówko, Nowogard, Poland) utilizing unsexed Ross 308 broilers purchased from a hatchery (Park Drobiarski Sp. z o.o., Śmiłowo, Kaczory, Poland). All procedures were overseen by the local veterinary authority (Goleniów) to ensure animal welfare compliance. Upon arrival, one-day-old chicks were systematically randomized into three distinct treatment cohorts (*n* = 100 per group) by farm personnel who were unaware of the study group assignments to prevent bias. The birds were housed in a controlled environment within separate pen areas. Chickens in the control group (C) were raised on a standard diet without any additive. The two treatment groups (H and HE) had their feed supplemented with hydrogel capsules for the first ten days of life. The capsules were offered ad libitum on chick paper placed under the water lines; its rustling sound attracted chicks and promoted feeding and drinking. The H group was given plain hydrogel alginate capsules without EO, while the HE group received capsules containing immobilized LEO (0.4 mL/L). Capsules were provided twice daily (8:00 and 18:00) in 2 kg portions distributed evenly across four points in each pen to ensure uniform access. The dose included a 10% overage to account for potential loss. All capsules were ingested during the 10-day supplementation period (days 1–10). Pens were inspected every 2 h during daylight to record consumption patterns and behavior. Birds had unrestricted access to water throughout the trial. All chickens were kept together for 35 days on wheat straw bedding at a density of 14 birds/m2. Environmental conditions followed Ross 308 guidelines [37], with temperatures gradually reduced from 32–33 °C initially to approximately 22 °C by day 41. Relative humidity increased progressively from 50% to 70% during the trial. Birds received commercial feed (Polskie Zakłady Zbożowe Sp. z o.o., Wałcz, Poland) ad libitum appropriate for each growth phase: starter (days 1–10), grower I (days 11–20), grower II (days 21–30), and finisher (days 31–35). Detailed feed composition and nutrient profiles are presented in Supplementary Table S1. On day 35, ten birds from each group were humanely slaughtered by decapitation. Breast muscle samples were collected and immediately stored at −82 °C for analyses. All procedures complied with Polish and EU regulations on animal experimentation (Directive 2010/63/EU) and adhered to institutional ethical standards. The study was carried out under veterinary supervision in a secure facility, ensuring animal welfare and preventing distress or harm.

### 2.3. Chemical Composition Analysis

The basic chemical composition of the breast muscle samples was determined using standardized analytical methods, with all gravimetric measurements performed using an Entris 224I-1S analytical balance (Sartorius, Goettingen, Germany). Water content was quantified following drying to constant weight in a DRY-Line 115 laboratory oven (VWR, Quito, Ecuador). Protein content was determined by the Kjeldahl method through sample mineralization (K-435 Digestion Unit, Büchi, Flawil, Switzerland), nitrogen distillation (KjelFlex K-360 apparatus, Büchi), and titration, with crude protein content calculated from nitrogen content using standard conversion factors. Fat content was determined via Soxhlet extraction of 3.0 g samples (weighed to 0.0001 g accuracy), involving 12 h diethyl ether (Chempur, Piekary Śląskie, Poland) extraction, solvent evaporation under a fume hood, and final drying at 105 °C to constant weight before mass difference calculation. Ash content was measured following incineration in a FCF 22SP laboratory furnace (CZYLOK, Jastrzebie-Zdrój, Poland).

### 2.4. Determination of Oxidative Stress Markers

Breast muscle samples (0.3 g) were homogenized in 3 mL of ice-cold 0.9% NaCl and centrifuged (12,850× *g*, 10 min, 4 °C), with the resulting supernatant aliquoted and stored at −80 °C until analysis. Lipid peroxidation was assessed by measuring the concentration of thiobarbituric acid reactive substances (TBARS) according to Chodkowska et al. [38]. Briefly, 100 μL of the supernatant was boiled for 60 min with 100 μL of 15% trichloroacetic acid and 100 μL of 0.37% thiobarbituric acid. After cooling to room temperature, the samples were centrifuged (10,000× *g*, 10 min), and the absorbance was read at 532 nm using a SpectraMax iD3 microplate reader (Molecular Devices, San Jose, CA, USA) and quantified against a malondialdehyde standard curve. Catalase activity in muscle supernatants was analyzed photometrically using the Catalase Assay Kit (Megazyme, Bray, Ireland), and glutathione peroxidase was determined using the Glutathione Peroxidase (GSH-PX) Assay Kit (BT LAB, Shanghai, China). Total superoxide dismutase (SOD) activity in breast muscle was measured spectrophotometrically based on the inhibition of pyrogallol autooxidation at alkaline pH, according to a microplate method described by Wypych et al. [39]. Differentiation between CuZn SOD (cytosolic) and Mn SOD (mitochondrial) activities was carried out using 1 mM KCN, an inhibitor of cytosolic SOD. Total protein concentration in muscle supernatants was measured photometrically using the Bio-Rad Protein Assay Kit II (Bio-Rad, Hercules, CA, USA). All absorbance readings were performed using a SpectraMax iD3 microplate reader.

### 2.5. Determination of Fatty Acid Profile in Breast Muscles Using Gas Chromatography–Mass Spectrometry (GC-MS)

Briefly, frozen samples of breast muscle (~1.0 g) were placed in 7.5 mL amber glass vials with Teflon-lined caps (Supelco, Bellefonte, PA, USA). Fatty acids were analyzed in the form of methyl esters (FAMEs) obtained from samples as described in the PN-EN ISO 12966-2:2017-05 [40] standard—boron trifluoride (BF_3_) method. FAMEs were analyzed using GC-MS system (Clarus 600 with Clarus 600T detector, PerkinElmer, Waltham, MA, USA) equipped with a TC-80 capillary column (60 m × 0.25 mm, 0.25 µm film; GL Sciences Inc., Tokyo, Japan). Helium (purity 6.0) was used as the carrier gas at 1 mL/min. Samples (1 μL) were injected in split mode (50:1) at 200 °C. The column temperature program consisted of an initial hold at 110 °C for 5 min, ramping at 5 °C/min to 180 °C (15 min hold), followed by 5 °C/min to 290 °C (5 min hold). The transfer line was maintained at 290 °C. Mass spectra were recorded in positive electron ionization mode (70 eV) using TurboMass software (v1.2). Identification of individual FAMEs relied on retention times, molecular ion confirmation, and spectral matching against the NIST 02 library, supported by a commercial reference mix containing 37 fatty acids (C4–C24; Sigma-Aldrich, St. Louis, MO, USA). Each sample was analyzed in triplicate (Supplementary Figure S7).

### 2.6. Determination of Mineral Content in Breast Muscles

Selected minerals (P, K, Ca, Na, Mg, Zn, Si, Fe, Cu, Ba, Sr, Mn, Al, Se, Cr, Pb, and Cd) in breast muscles were quantified by inductively coupled plasma optical emission spectrometry (ICP-OES) using an Optima 2000DV instrument (Perkin Elmer, Waltham, MA, USA). Frozen samples (700 mg) were microwave-digested using a Speedwave Xpert oven (Berghof GmbH, Eningen, Germany) with 9.0 mL of 69% HNO_3_ and 1.0 mL of 30% H_2_O_2_ (Supelco, Bellefonte, USA) for 15 min before sealing. Calibration standards (0.1–150 mg/L) were prepared from a TraceCert^®^ multi-element standard solution (Sigma-Aldrich, St. Louis, USA) with yttrium (Merck, Darmstadt, Germany) applied as internal standard. Mineralized samples were diluted with demineralized water, 4.0 mL of 69% HNO_3_, yttrium solution and spectral buffer. Calibration curves showed R^2^ > 0.99; mean recovery was 96–98%. All analyses were performed in triplicate, with quality control utilizing certified bovine muscle reference material (NIST 8414; Gaithersburg, MD, USA) (Supplementary Table S3).

### 2.7. Statistical Analysis

All statistical analyses were performed using SAS software (version 9.4, 2012; SAS Institute, Cary, NC, USA). Individual pens were considered replicates, with each replicate representing an experimental unit. The obtained results of chemical composition, oxidative stress markers, fatty acid profile, and mineral contents of breast muscles were subjected to statistical analysis, calculating the means and standard error of mean (SEM). Data normality was assessed using the Shapiro–Wilk test. Differences between groups were evaluated using the Kruskal–Wallis test, followed by Dunn’s pairwise comparisons. Statistical significance was set at *p* ≤ 0.05.

## 3. Results

### 3.1. Chemical Composition of Lavender Essential Oil (LEO)

The chemical composition data of the LEO was published in a previous study [2]. The main chemical components identified by GC-MS analysis were: linalyl acetate (41.20%), linalool (31.55%), 4-terpineol (2.05%), (Z)-β-ocimene (2.70%), and β-caryophyllene (2.59%).

### 3.2. Determination of Basic Chemical Composition

Table 1 presents the basic chemical composition of broiler chicken breast muscles. A significant reduction in dry matter content was observed in the HE group, accompanied by an increase in water content (*p* < 0.05). No significant differences were found in protein, fat, or ash contents (*p* > 0.05).

### 3.3. Determination of Oxidative Stress Markers

The effects of feed additives on oxidative stress markers in breast muscle are summarized in Table 2. Feeding the H and HE diets did not affect TBARS concentration or catalase and glutathione peroxidase activities in chicken breast muscle. Broilers fed the HE diet had lower total SOD (*p* < 0.01) and CuZn SOD (*p* < 0.05) activities in the muscle, while Mn SOD activity was lower (*p* < 0.05) in birds receiving the H diet compared to the C group.

### 3.4. Determination of Fatty Acid Profile

Table 3 summarizes the fatty acid profile of breast muscles from 35-day-old broiler chickens. The muscles were characterized by the highest average content of palmitic acid (C16:0)—24.6%; stearic acid (C18:0)—9.85%; oleic acid (C18:1n9c)—33.17%; and linoleic acid (LA—C18:2n6c)—16.6%.

Dietary supplementation with hydrogel capsules significantly affected the contents of myristic acid (C14:0), palmitic acid (C16:0), margaric acid (C17:0), oleic acid (C18:1n9c), linoleic acid (C18:2n6c), and dihomo-γ-linolenic acid (C20:3n6). However, no statistical differences were observed in their levels in relation to the type of supplement tested. A significant (*p* ≤ 0.05) value was found for PUFAs, n3 fatty acids, and the n6/n3 ratio. In addition, breast muscles of the HE birds contained lower SFA levels compared to the control group (C) (*p* ≤ 0.05). On average, breast muscles contained 35.57% SFAs, 41.03% MUFAs, and 23.4% PUFAs. The highest and most significantly different n6/n3 ratio was found in the control group (C) (*p* < 0.05). No significant differences were observed in the MUFA/PUFA and PUFA/SFA ratios (*p* > 0.05); however, differences were found in the n6/n3 and MUFA/SFA ratios (*p* < 0.05).

### 3.5. Determination of Mineral Composition in Breast Muscles

The results of the mineral composition analysis of the breast muscles of broiler chickens are presented in Table 4. The average concentrations of micronutrients were as follows: Zn, 8.3–9.0 mg/kg; Si, 4.56–5.77 mg/kg; Fe, 4.59–5.46 mg/kg; Cu, 0.32–0.41 mg/kg; Ba, 0.03–0.04 mg/kg; Mn, 0.16–0.18 mg/kg; Al, 0.23–0.30 mg/kg; Se, 3.04–3.40 mg/kg; Cr, 0.21–0.23 mg/kg; Pb, 0.089–0.090 mg/kg; and Cd, 0.042–0.049 mg/kg. The average concentrations of macronutrients were as follows: P, 3148–3238 mg/kg; K, 4496–4954 mg/kg; Ca, 57.3–66.0 mg/kg; Na, 586–731 mg/kg; and Mg, 381–392 mg/kg.

## 4. Discussion

Broiler chicken meat is considered a dietetic product due to its low intramuscular fat content and high protein proportion [41,42]. The quality and nutritional value of poultry meat are primarily determined by its basic chemical composition. The study did not reveal any statistically significant differences between groups in the content of these components, except for significantly (*p* ≤ 0.05) higher ash and water levels in the HE group. The proportions of basic chemical constituents influence the culinary and technological value of meat. The breast muscles of broiler chickens were characterized by a high protein content, averaging 23.69%, and a low fat content of 1.18%. The basic chemical composition of the breast muscles determined in the present study is consistent with findings reported by other authors [41,43]. Lipid oxidation, induced by reactive oxygen species, is a major factor contributing to the deterioration of food quality and the formation of compounds potentially harmful to human health. The use of antioxidants to delay rancidity is a common strategy to extend the shelf life of meat products. However, the application of synthetic antioxidants in food, such as BHT (butylated hydroxytoluene) or BHA (butylated hydroxyanisole), raises increasing concerns due to reports of their potential carcinogenic properties [44]. Moreover, growing consumer demand for natural products free from synthetic additives has intensified interest in natural compounds with antioxidant activity, including EOs.

The mechanisms by which EOs affect meat oxidative stability and other parameters are complex and multifactorial. The main factor responsible for their antioxidant properties is the abundance of bioactive compounds, such as terpenoids [9,45]. LEO is a mixture of volatile organic compounds from the group of terpenoids [6]. These compounds can neutralize free radicals by donating hydrogen atoms or electrons, thereby interrupting the chain reactions of lipid oxidation [46]. Additionally, many EOs can chelate transition metal ions, such as iron and copper, which are strong pro-oxidants and catalyze the formation of free radicals. Moreover, EOs may influence meat oxidative stability by modulating the intestinal microflora of animals. A healthy intestinal microbiota can improve nutrient utilization and reduce the production of toxins that may impact the body’s antioxidant system [47]. Active compounds in essential oils act as digestive enhancers, balance the intestinal microbial ecosystem, and stimulate the secretion of endogenous digestive enzymes, potentially improving poultry growth performance. Our previous studies have demonstrated that LEO positively affects production parameters and microbiota in broiler chickens [6,48]. The antioxidant activity of LEO can be attributed to the presence of linalool and linalyl acetate, its main components, which show antioxidant properties [45]. Their presence in the diet of broiler chickens may have strengthened the birds’ endogenous antioxidant system, resulting in reduced SOD activity, which may indicate a lower demand for this enzyme under decreased oxidative stress conditions.

Appropriate biomarkers reflecting damage to key cellular macromolecules are used to monitor the effectiveness of the antioxidants. Malondialdehyde (MDA), measured based on its reaction with thiobarbituric acid, is a commonly used marker of lipid peroxidation. Since other compounds formed during peroxidation also react with this reagent, lipid peroxidation levels are often expressed as thiobarbituric acid reactive substances (TBARSs). This method is appropriate for experimental settings focused mainly on detecting differences in this parameter between animal groups, rather than measuring the absolute levels of lipid peroxidation products [49]. The enzymatic barrier that protects the body from the harmful effects of reactive oxygen species consists of several enzymes. This group primarily includes superoxide dismutase (SOD), catalase (CAT), and glutathione peroxidase (GPx). Complete antioxidant protection depends on the synergistic interaction of all enzymes. The imbalance between reactive oxygen species production and the capacity of the antioxidant defense system results in increased intensity of lipid peroxidation and reduced activity of SOD, catalase and glutathione peroxidase [50,51,52,53]. Considering that catalase and glutathione peroxidase activities, as well as TBARS concentration, were not affected by feeding the H or HE diet, it can be concluded that dietary supplementation did not induce oxidative stress in the breast muscle of chickens. LEO is a rich source of antioxidants [54,55]. However, the present study found that dietary supplementation with HE reduced total SOD activity in the breast muscle of broiler chickens by decreasing CuZn SOD activity. This enzyme is encoded by the *SOD1* gene and is present mostly in intracellular cytoplasmic spaces [56]. Cu and Zn are trace elements necessary for the activity of SOD1 because they are present in its catalytic center [56]. The lower CuZn SOD activity in the breast muscle of broilers fed a diet supplemented with HE likely resulted from reduced Cu content in the muscle. However, feeding the HE diet also decreased Mn concentration in the muscle but did not affect the activity of Mn SOD, the mitochondrial isoform of the enzyme. Surprisingly, Mn SOD activity was lower in the H group than in the control, although no difference in Mn concentration in the muscle was observed between these groups. The results indicate that HE supplementation may downregulate *SOD1* expression in breast muscles, but further research is required to confirm this assumption. In contrast, the experiment by Küçükyilmaz et al. [11] showed that breast muscles of chickens fed a diet supplemented with essential oil (*Lavandula stoechas*) at concentrations of 24 and 48 mg/kg had higher superoxide dismutase (SOD) levels compared to birds that did not receive EO supplementation. Other EOs also demonstrate the ability to improve meat oxidative stability [57,58,59]. For example, He et al. [57] showed that supplementation with oregano EO improved meat quality, antioxidant capacity, and nutritional value in cattle meat. Similarly, studies on bamboo essential oil suggest its potential use as a natural preservative in meat products, improving their oxidative stability and shelf life [58]. In the context of poultry meat, Özbilgin et al. [60] also investigated the effect of LEO on meat quality and antioxidant status in quail and reported improvements in both areas. These studies confirm that EOs have the potential to support natural antioxidant mechanisms in livestock, which contributes to improved meat quality and shelf life.

Compared to meat from other animal species, poultry meat contains relatively little fat, which determines its high dietary and nutritional value. At the same time, nearly 70% of the lipids in poultry meat are unsaturated fatty acids, including essential unsaturated fatty acids (EUFAs) such as linoleic acid at approximately 16 g/100 g of fat and α-linolenic acid at 3 g/100 g of fat [61]. Lipid peroxidation is a free radical-driven oxidation process affecting polyunsaturated fatty acid residues in phospholipids, resulting in the formation of lipid hydroperoxides. The mechanism of this process consists of three stages: initiation, propagation, and termination. During the initiation stage, a molecule of acid is converted into a free alkyl radical under the influence of a hydroxyl, peroxyl, alkoxyl, or alkyl radical. During propagation, alkyl radicals react with oxygen to form peroxyl radicals, which can extract hydrogen atoms from additional polyunsaturated fatty acid molecules. The reaction cycle can repeat multiple times until all substrates are depleted or termination occurs, potentially leading to the formation of hydroperoxides from several hundred polyunsaturated fatty acid molecules, depending on the frequency of termination reactions. Termination is a reaction between free radicals that produces a non-radical product. Termination products include dimers of fatty acids as well as keto- and hydroxy fatty acids, which are modified, damaged lipid molecules [52]. In the cell, peroxidation takes place in membranes containing proteins, which react with free radicals to form protein radicals. These in turn can participate in termination reactions, creating mixed protein–lipid complexes. Reinitiation can occur, particularly when non-radical peroxidation products (lipid hydroperoxides) decompose, generating alkoxyl radicals and thus forming free radicals again. The fatty acid profile in poultry meat is crucial for its nutritional and health-promoting value. GC–MS was used to analyse the FAMEs composition of breast muscles (Table 3). The highest proportions in all breast muscle samples were C16:0 and C18:1n9c. FAMEs have characteristic fragmentation patterns due to the specific way in which the fatty acid breaks down, and how it undergoes McLafferty rearrangement to produce new ions. The parent ion is the molecular ion, which is equal to the molecular mass, and this peak is used as a reference to further characterize other peaks [62]. The mass spectra of all saturated FAMEs have a base peak at *m*/*z* 74, which is due to a McLerffaty rearrangement (Supplementary Materials Figure S14). Other peaks are those observed at *m*/*z* 227, which is due to a loss of hydrogen atom and a propyl radical [M–43]^+^, and also *m*/*z* 239, occurring as a result of α-cleavage of methoxy group [M–31]^+^ [62,63]. The other distinct peaks observed for saturated fatty acid were those at *m*/*z* 87, 115, 129, 143, 157, 171, 199, 213 and 227, with each having a difference of 14 *amu*. These ions were formed due to β-cleavage of carbomethoxy ion [63]. Similarly, palmitic acid methyl ester (C16:0) could be identified with peaks at *m*/*z* 270, 227 and 199 (Supplementary Materials Figures S12 and S13). The monounsaturated FAME identified in breast muscles samples was oleic acid (C18:1n9c) with a relative percentage of 33.1% –33.2%. The base peak for this FAMEs is at *m*/*z* 55; other peaks are those observed at *m*/*z* 222 and 264 occurring due to loss of McLafferty ion [M–74] and a methoxy group [M–32]^+^ plus hydrogen ion (Supplementary materials Figures S8 and S9) [63]. Analysis of the experimental results showed that supplementation with hydrogel immobilizing LEO had a positive effect on the lipid composition of breast muscles. The study observed a significant reduction in SFA content in the HE group compared to the control group. This change is beneficial for consumer health, as excessive intake of SFAs is associated with an increased risk of cardiovascular diseases. At the same time, an improvement in the n6/n3 ratio was observed, with the lowest value recorded in the HE group, which was desirable due to its health benefits. Changes in the fatty acid profile induced by EOs have also been observed by other authors [14,57]. Amer et al. [14] demonstrated an effect on the fatty acid profile, but in a slightly different trend. The authors observed a significant, dose-dependent increase in the content of omega-3 (n-3 PUFAs) and omega-6 (n-6 PUFAs), along with an improved n-3/n-6 ratio. This difference in the observed trends may result from differences in the EO administration methodology and duration of its application. On the other hand, He et al. [57], in a study on cattle, observed that supplementation with oregano EO influenced the fatty acid profile, suggesting broader applications of EOs in modifying meat lipid composition. Mechanisms responsible for modifying the fatty acid profile under the influence of EOs may include effects on the activity of enzymes involved in lipid synthesis and metabolism [64]. Active components of LEO, such as linalool and linalyl acetate, may affect the expression of genes encoding desaturase and elongase enzymes, which are key to the synthesis of PUFAs. Additionally, the antioxidant properties of LEO may protect PUFAs from peroxidation, contributing to their preservation at higher levels in tissues. This is especially important for omega-3 fatty acids, which are particularly susceptible to oxidation due to their chemical structure [65].

Analysis of the mineral composition of broiler chicken breast muscles in this study revealed significant changes in the content of certain minerals. The HE group showed significantly higher sodium content but lower levels of copper, iron, manganese, and aluminium compared to the control group (*p* < 0.05). These changes are important because many of these elements play key roles in the body, acting as cofactors for antioxidant enzymes (e.g., selenium, zinc, copper, and manganese) and participating in numerous metabolic processes. The reduction in copper content in the HE group is particularly notable in light of decreased CuZn SOD activity, since copper is an essential component of this enzyme. This may suggest that the hydrogel with immobilized LEO affects copper bioavailability or metabolism in chickens. Similarly, the reduction in iron content, which is also involved in oxidative processes, may affect the overall antioxidant status of the organism. Conversely, the increased sodium content in the breast muscles of the HE group could be related to the composition of the alginate hydrogel, which contains this element. This may also indicate alterations in the body’s water–electrolyte balance [66]. The results of studies by other authors on the use of phytogenic feed additives and their impact on the mineral composition of chicken breast meat are inconsistent [53,67]. This may indicate that the effect of EOs on mineral composition is specific to the particular oil and its chemical composition, as well as to individual elements. Differences in results may also be related to interactions between dietary components and EOs, as well as the overall health status and metabolism of the birds. Further research is needed to fully understand the mechanisms by which EOs affect mineral absorption and distribution in chicken tissues.

## 5. Conclusions

The present results may contribute to the development of new nutritional strategies in poultry production. Immobilizing LEO in alginate hydrogels may represent an innovative strategy that affects the bioavailability of active components. The use of hydrogel capsules with immobilized essential oil as a dietary supplement for broiler chickens has the potential to improve meat quality, particularly by modifying the fatty acid profile toward a healthier composition. Favorable modifications of the fatty acid profile were observed, including a reduced content of saturated fatty acids and an improved n6/n3 ratio, which are desirable from a consumer health perspective. No significant differences were observed in TBARS levels, suggesting that the overall oxidative stability of the meat was not compromised and that antioxidant mechanisms are complex and may involve other pathways. The reduced activity of CuZn-SOD may be linked to the decreased copper content in the muscle tissue. Supplementation affected the mineral composition of the muscles, and changes may indicate an effect of the dietary supplement on mineral metabolism and bioavailability. Further comprehensive studies are necessary to fully understand the mechanisms of action of hydrogel with immobilized LEO. Further research should include optimization of its application in poultry production (doses, essential oil concentrations, duration of administration, and different commercial chicken broiler lines) to achieve maximum health benefits for both animals and consumers of poultry meat.

## Figures and Tables

**Table 1:** Chemical composition of broiler chicken breast muscle.

Item	Group ^1^				*p*-Value ^2^		
	C	H	HE	SEM	C vs. H	H vs. HE	HE vs. C
Dry matter	23.7	23.5	22.8	0.14	0.172	0.001	0.007
Water	76.3	76.5	77.2	0.14	0.172	0.001	0.007
Protein	24.1	23.9	23.8	0.45	0.437	0.480	0.322
Fat	1.18	1.28	1.15	0.14	0.599	0.522	0.611
Ash	1.20	1.21	1.16	0.01	0.551	0.624	0.888

^1^ C: Control; H: Hydrogel; Hydrogel + LEO. ^2^ Kruskal–Wallis test, Statistical significance is set at *p* ≤ 0.05.

**Table 2:** Effects of hydrogel addition on oxidative stress markers in broiler chicken breast muscles.

Item	Group ^1^				*p*-Value ^2^		
	C	H	HE	SEM	C vs. H	H vs. HE	HE vs. C
TBARS ^3^, μM/g	12.2	9.8	10.2	1.02	0.465	1.000	0.560
Catalase, U/mg protein	3.6	3.8	3.1	0.18	1.000	0.312	0.791
Total SOD ^4^, U/mg protein	85.5	70.2	58.3	3.65	0.252	0.465	0.005
Mn SOD ^4^, U/mg protein	38.7	26.3	32.8	1.79	0.016	0.560	0.422
CuZn SOD ^4^, U/mg protein	46.8	43.9	25.5	3.54	1.000	0.126	0.013
Glutathione peroxidase, U/mg protein	17.0	10.9	10.6	1.86	1.000	1.000	1.000

^1^ C: Control; H: Hydrogel; Hydrogel + EO. ^2^ Kruskal–Wallis test, Statistical significance is set at *p* ≤ 0.05. ^3^ TBARS—Thiobarbituric acid reactive substances. ^4^ SOD—Superoxide dismutase.

**Table 3:** Effect of hydrogels on fatty acid profile (%) and quantitative content (mg/100 g) * in breast muscles.

Item	Group ^1^				*p*-Value ^2^		
	C	H	HE	SEM	C vs. H	H vs. HE	HE vs. C
C14:0	0.95 0.106 *	1.06 0.129 *	0.97 0.106 *	0.09	0.041	0.066	0.711
C16:0	24.8 2.78 *	24.4 2.967 *	24.6 2.688 *	0.29	0.025	0.173	0.246
C17:0	0.14 0.016 *	0.18 0.022 *	0.12 0.013 *	0.03	0.003	0.0001	0.032
C18:0	9.96 1.117 *	9.97 1.212 *	9.62 1.051 *	0.30	0.954	0.070	0.066
C14:1	0.16 0.018 *	0.15 0.018 *	0.16 0.017 *	0.002	0.270	0.412	0.722
C16:1	5.54 0.621 *	5.75 0.699 *	5.62 0.614 *	0.04	0.062	0.192	0.457
C17:1	0.32 0.036 *	0.33 0.04 *	0.33 0.036 *	0.01	0.535	0.903	0.595
C18:1 n9t	1.19 0.133 *	1.27 0.154 *	1.15 0.126 *	0.02	0.035	0.005	0.245
C18:1 n9c	33.1 3.711 *	33.2 4.037 *	33.2 3.627 *	0.04	0.489	0.557	0.234
C20:1 n9	0.45 0.05 *	0.43 0.052 *	0.39 0.043 *	0.01	0.497	0.145	0.052
C22:1 n9	0.11 0.012 *	0.11 0.013 *	0.12 0.013 *	0.002	0.577	0.198	0.407
C18:2 n6c	16.6 1.86 *	16.5 2.006 *	16.8 1.835 *	0.07	0.420	0.049	0.172
C18:3 n6	0.15 0.017 *	0.15 0.018 *	0.15 0.016 *	0.002	0.368	0.227	0.695
C18:3 n3	1.57 0.176 *	1.63 0.198 *	1.70 0.186 *	0.03	0.465	0.349	0.125
C20:2 n6	0.29 0.033 *	0.33 0.04 *	0.32 0.035 *	0.01	0.128	0.764	0.184
C20:3 n6	0.31 0.035 *	0.34 0.041 *	0.24 0.026 *	0.01	0.326	0.005	0.028
C20:3 n3	0.18 0.02 *	0.18 0.022 *	0.17 0.019 *	0.003	0.837	0.111	0.137
C20:4 n6	1.56 0.175 *	1.44 0.175 *	1.49 0.163 *	0.03	0.163	0.560	0.356
C20:5 n3	0.65 0.073 *	0.60 0.073 *	0.72 0.079 *	0.02	0.291	0.046	0.245
C22:6 n3	1.94 0.217 *	2.03 0.247 *	2.07 0.226 *	0.03	0.174	0.580	0.079
SFA ^3^	35.8	35.6	35.3	0.08	0.110	0.072	0.004
MUFA ^4^	40.9	41.2	41.0	0.07	0.071	0.189	0.508
PUFA ^5^	23.3	23.2	23.7	0.09	0.699	0.037	0.061
n3	4.35	4.44	4.66	0.04	0.038	0.0003	0.0001
n6	18.9	18.7	19.0	0.08	0.382	0.197	0.608
MUFA ^4^/SFA ^3^	1.14	1.16	1.16	0.01	0.028	0.667	0.015
MUFA ^4^/PUFA ^5^	1.76	1.78	1.73	0.01	0.361	0.052	0.216
PUFA ^5^/SFA ^3^	0.63	0.65	0.67	0.01	0.304	0.266	0.053
n6/n3	4.35	4.22	4.08	0.04	0.035	0.026	0.001

^1^ C: Control; H: Hydrogel; Hydrogel + LEO. ^2^ Kruskal–Wallis test, Statistical significance is set at *p* ≤ 0.05. ^3^ SFA—Saturated fatty acids. ^4^ MUFA—Monounsaturated fatty acids. ^5^ PUFA—Polyunsaturated fatty acids.

**Table 4:** Mineral contents of breast muscles in 35-day-old broiler chickens.

Item	Group ^1^				*p*-Value ^2^		
	C	H	HE	SEM	C vs. H	H vs. HE	HE vs. C
Mg	392	381	384	3.71	0.246	0.730	0.372
Ca	57.3	64.7	66.0	2.41	0.218	0.828	0.173
Na	586	666	731	17.8	0.031	0.076	0.001
K	4954	4720	4496	60.2	0.073	0.084	0.002
Zn	8.3	8.8	9.0	0.16	0.239	0.531	0.092
Cu	0.37	0.41	0.32	0.01	0.104	0.001	0.041
Fe	5.05	5.46	4.59	0.10	0.050	0.0003	0.034
Mn	0.18	0.17	0.16	0.003	0.260	0.122	0.014
Cr	0.22	0.21	0.23	0.004	0.800	0.121	0.163
Pb	0.089	0.087	0.090	0.001	0.628	0.399	0.679
Cd	0.042	0.043	0.049	0.001	0.909	0.070	0.068
Se	3.34	3.40	3.04	0.10	0.801	0.176	0.235
P	3175	3148	3238	27.2	0.692	0.212	0.350
Al	030	0.27	0.23	0.01	0.299	0.123	0.017
Ba	0.039	0.033	0.040	0.002	0.123	0.104	0.856
Si	5.77	4.56	5.19	0.22	0.034	0.232	0.275

^1^ C: Control; H: Hydrogel; Hydrogel + LEO. ^2^ Kruskal–Wallis test, Statistical significance is set at *p* ≤ 0.05.

## Data Availability

The original contributions presented in this study are included in the article. Further inquiries can be directed to the corresponding author.

## Supplementary Materials

The following supporting information can be downloaded at https://www.mdpi.com/article/10.3390/foods14193409/s1, Table S1. Ingredient and nutrient composition of the basal diets; Table S2. Selected abundances (m/z*) and retention time (RT) for fatty acid methyl ester (FAME) GC-MS analysis; Table S3. Reference material—bovine muscle 8414; Figure S1. Total Ion Chromatogram of *Lavandula angustifolia* essential oil obtained by GC-MS method; Figure S2. Mass spectrum of linalool present in *Lavandula angustifolia* essential oil, compared with linalool standard mass spectrum from NIST 02 library; Figure S3. Mass spectrum of linalool acetate present in *Lavandula angustifolia* essential oil, compared with linalool acetate standard mass spectrum from NIST 02 library; Figure S4. Mass spectrum of 4-terpineol present in *Lavandula angustifolia* essential oil, compared with 4-terpineol standard mass spectrum from NIST 02 library; Figure S5. Mass spectrum of lavandulyl acetate present in *Lavandula angustifolia* essential oil, compared with lavandulyl acetate standard mass spectrum from NIST 02 library: Figure S6. Mass spectrum of caryophyllene present in *Lavandula angustifolia* essential oil, compared with caryophyllene standard mass spectrum from NIST 02 library; Figure S7. Chromatogram of *F.A.M.E. MIX C4-C24* (references material) obtained by GC-MS method; Figure S8. Mass spectrum of oleic acid methyl ester (C18:1 n9c) present in breast muscle of broiler chicken, compared with mass spectrum in references material FAMEs mixture; Figure S9. Standard mass spectrum of oleic acid methyl ester (C18:1 n9c) from NIST 02 library; Figure S10. Mass spectrum of elaidic acid methyl ester (C18:1 n9t) present in breast muscle of broiler chicken, compared with mass spectrum in references material FAMEs mixture; Figure S11. Standard mass spectrum of elaidic acid methyl ester (C18:1 n9t) from NIST 02 library; Figure S12. Mass spectrum of palmitic acid methyl ester (C16:0) present in breast muscle of broiler chicken, compared with mass spectrum in references material FAMEs mixture; Figure S13. Standard mass spectrum of palmitic acid methyl ester (C16:0) from NIST 02 library; Figure S14. Mass spectrum of palmitic acid methyl ester (C16:0).

## Abbreviations

The following abbreviations are used in this manuscript:

LEO	Lavender essential oil
CAT	Catalase
ROS	Reactive oxygen species
SOD	Superoxide dismutase
TBARSs	Thiobarbituric acid reactive substances
