# Supplementary material for: Impact of Dietary Alginate Hydrogel Capsules with Lavender Essential Oil on Oxidative Stability, Fatty Acid Profile, and Mineral Composition of Breast Muscles in Broiler Chickens
Source: Foods. 2025 Oct 2;14(19):3409. doi: 10.3390/foods14193409 (PMC12523348; doi:10.3390/foods14193409)

Supplementary material

Supplementary Table S1. Ingredient and nutrient composition of the basel diets (%)

Item	Starter (0–10 d)	Grower I (11–20 d)	Grower II (21–30 d)	Finisher (31–35 d)
Ingredient				
Wheat, 11.6%	37.00	38.00	33.00	40.00
Soybean meal, 46%	29.05	24.94	27.16	24.0
Maize, 8%	25.93	27.39	28.74	25.32
Soy oil	2.68	2.78	3.79	3.66
Canola meal, 32.5%	-	2.50	2.50	3.01
Potato protein, 73%	1.50	1.0	0.50	-
Limestone	1.27	0.85	0.78	0.67
Monocalcium phosphate	0.92	0.43	0.30	0.11
Vitamin and mineral premix[1]	0.53	0.53	0.60	0.59
Poultry fat	-	0.50	1.50	1.50
L-Lys-HCl	0.39	0.40	0.41	0.43
DL-Met	0.26	0.17	0.18	0.17
Salt	0.24	0.27	0.28	0.28
$NaHCO_3$	0.14	0.13	0.15	0.14
Thr	0.07	0.06	0.07	0.07
Choline chloride	-	0.03	0.02	0.03
Phytase premix[2]	0.02	0.02	0.02	0.02
Calculated analysis				
ME (kcal/kg)	2800.0	2865.0	2984.1	3020.0
Lys	1.26	1.16	1.15	1.07
Met	0.56	0.46	0.45	0.44
Ca	0.86	0.67	0.71	0.65
P	0.56	0.47	0.42	0.39
Na	0.15	0.15	0.15	0.15
Analyzed nutrient composition[3]				
CP	21.50	20.00	19.50	18.50
Crude fiber	2.60	2.50	2.60	3.00
Crude fat	5.80	6.40	6.60	7.00
Crude ash	5.40	4.70	4.20	4.00

[1]Vitamin-mineral premix contained the following per kilogram of diet: vitamin A, 9,000 IU; vitamin D3, 4,000 IU; vitamin E, 60 mg; vitamin B1, 3 mg; vitamin B2, 10 mg; vitamin B6, 3 mg; vitamin B12, 15 µg; nicotinic acid, 60 mg; pantothenic acid, 14.7 mg; folic acid, 1.5 mg; iron, 59.85 mg; copper, 15 mg; cobalt, 1.0 mg; zinc, 100 mg; iodine, 1.5 mg; selenium, 0.225 mg, antioxidant (BHA), 12.0 mg.

[2]Phytase premix was prepared by dilution with calcium carbonate to contain 1,500 FTU (phytase units)/g (Optiphos, Huvepharma AD, Sofia, Bulgaria)

[3]Based on a DM content of 87.5

Supplementary Table S2. Selected abundances (m/z*) and retention time (RT) for fatty acid methyl ester (FAME) GC-MS analysis. *Ion mass to charge ratio.

Compound	Retention time	Characteristic ions (m/z)
C14:0	28.05	74
C16:0	39.45	74
C17:0	43.13	74
C18:0	49.58	74
C14:1	46.06	55
C16:1	38.48	55
C17:1	42.43	55
C18:1 n-9t	45.56	296
C18:1 n-9c	45.35	55
C20:1 n-9	50.11	55
C22:1 n-9	53.85	55
C18:2 n-6c	44.15	294
C18:3 n-6	44.67	194
C18:3 n-3	45.15	292
C20:2 n-6	50.13	81
C20:3 n-6	49.99	67
C20:3 n-3	49.58	320
C20:4 n-6	49.17	318
C20:5 n-3	49.31	91
C22:6 n-3	52.95	342

Supplementary Table S3. Reference material - bovine muscle 8414

Element	Concentration of elements in reference material 8414 (mg/kg)					Fold change observed result/ certified result
	Certified result	1st replicate	2nd replicate	3rd replicate	Observed result	
Pb	0.38	0.37810	0.39030	0.3855	0.38460	1.0121
Cd	0.013	0.01412	0.01403	0.14001	0.01405	1.0807
Zn	142	144.9548	143.9961	141.5583	143.50306	1.0105
Cu	2.84	2.85051	2.83415	2.86014	2.48266	1.0029
Mn	0.37	0.39025	0.36531	0.38221	0.37925	1.0250
Cr	0,071	0.06804	0.069954	0.06889	0.068961	0.9712
Fe	71.2	69.71051	70.92395	74.00512	71.54652	1.0048
Al	1.7	1.66991	1.61889	1.68229	1.65703	0.9747
Ca	145	146.9942	149.07511	145.92551	147.33160	1.0160
Se	0.076	0.07271	0.069991	0.072991	0.0718973	0.9460
K	15170	15381.0	15221.50	15255.43	15318.215	1.0097
Na	2100	2055.150	2115.93	2075.95	2082.3433	0.9915
Mg	960	971.440	963.451	954.89	963.26033	1.0033
P	8360	8299.05	8315.991	8285.51	8300.1836	0.9928

Supplementary Figure S1. Total Ion Chromatogram of *Lavandula angustifolia* essential oil obtained by GC-MS method.

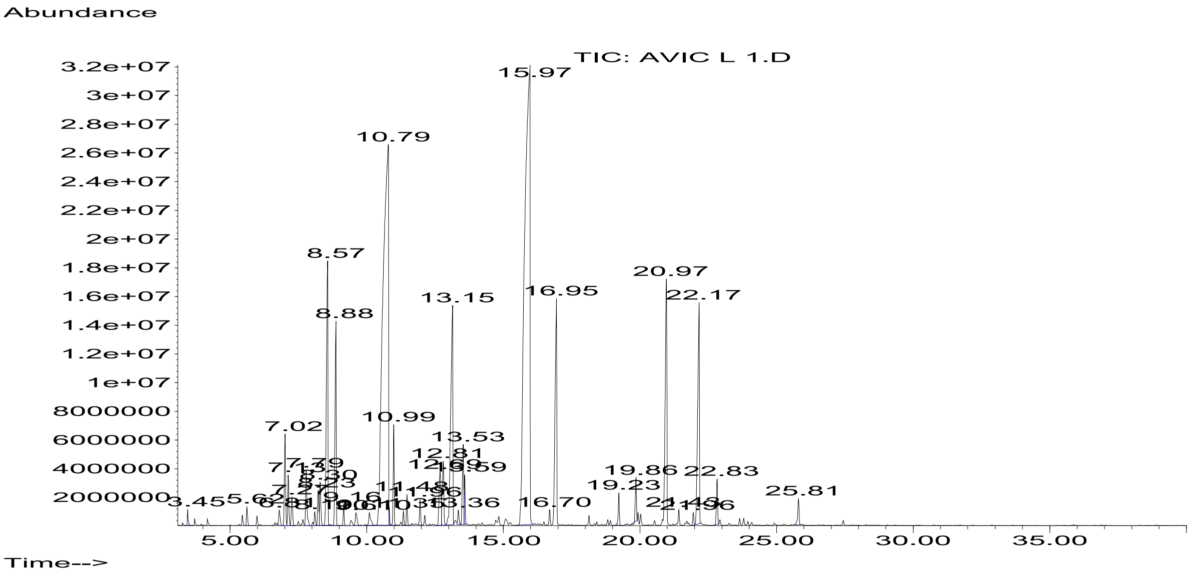

Supplementary Figure S2. Mass spectrum of linalool present in *Lavandula angustifolia* essential oil, compared with linalool standard mass spectrum from NIST 02 library.

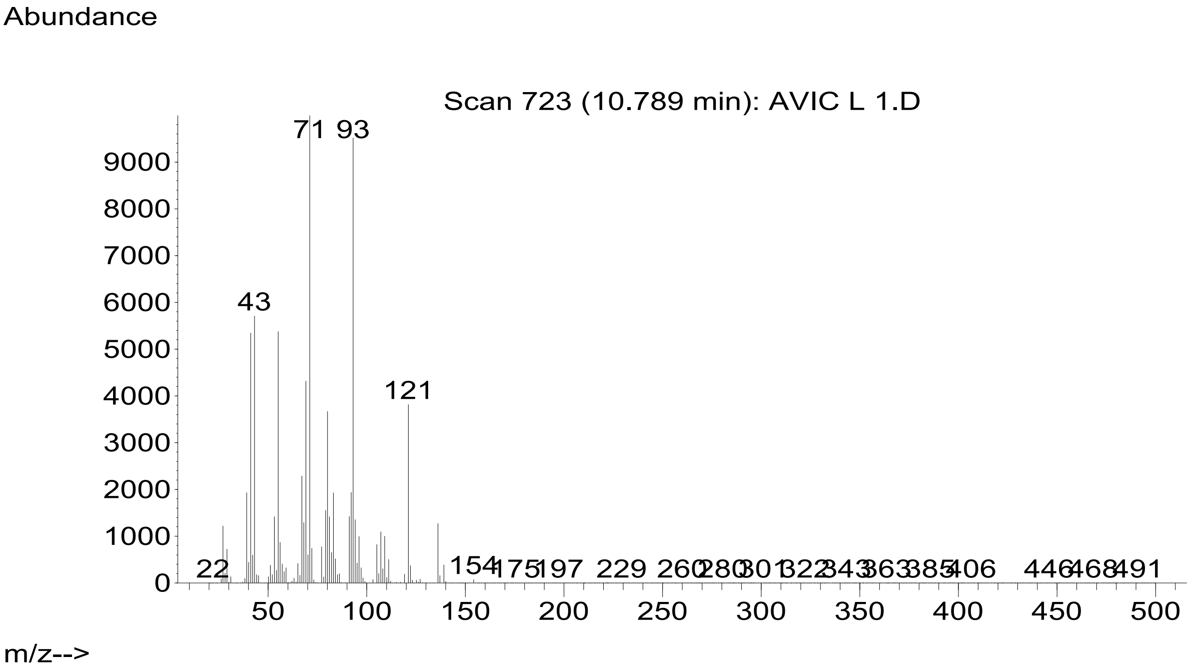

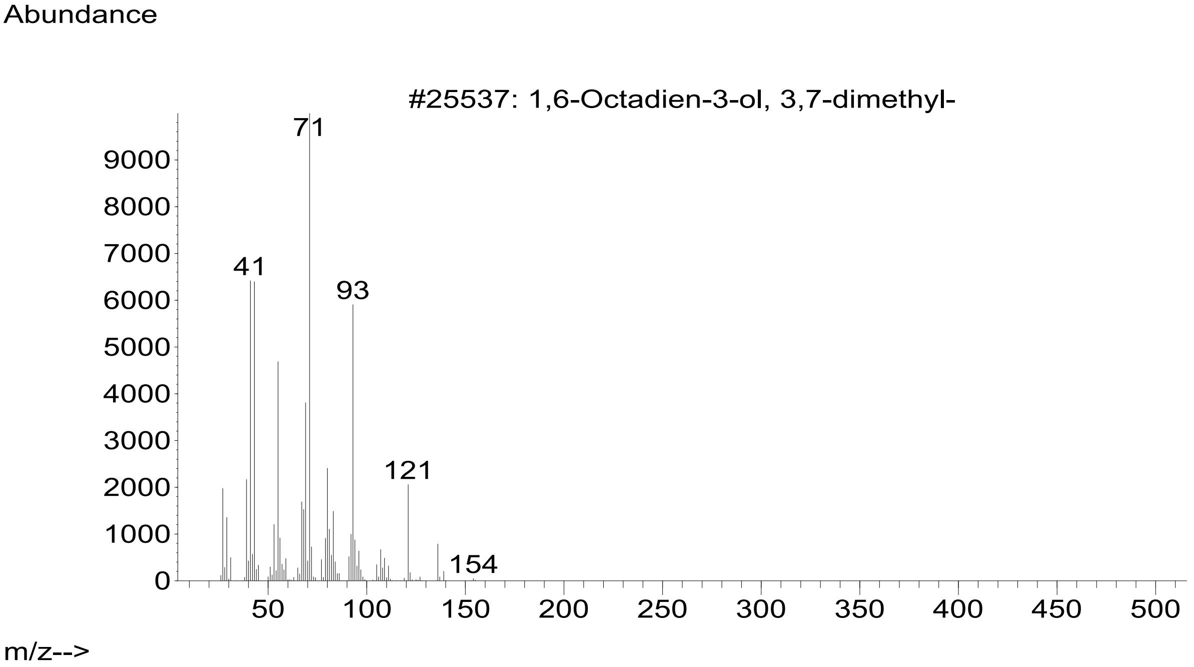

Supplementary Figure S3. Mass spectrum of linalool acetate present in *Lavandula angustifolia* essential oil, compared with linalool acetate standard mass spectrum from NIST 02 library.

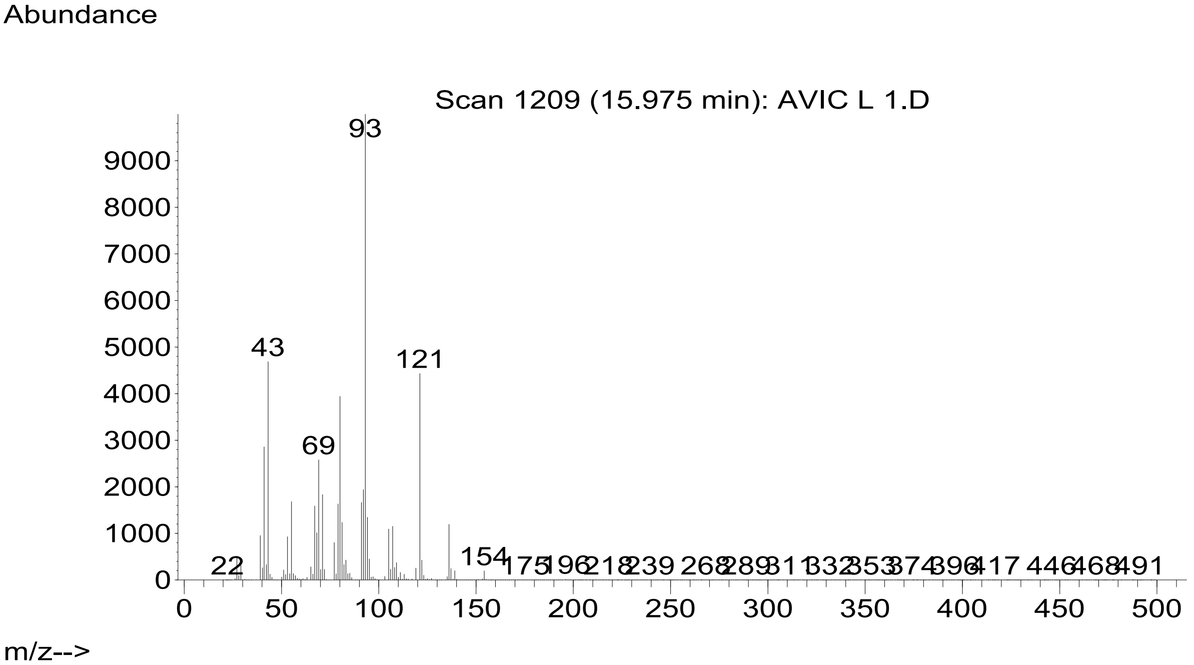

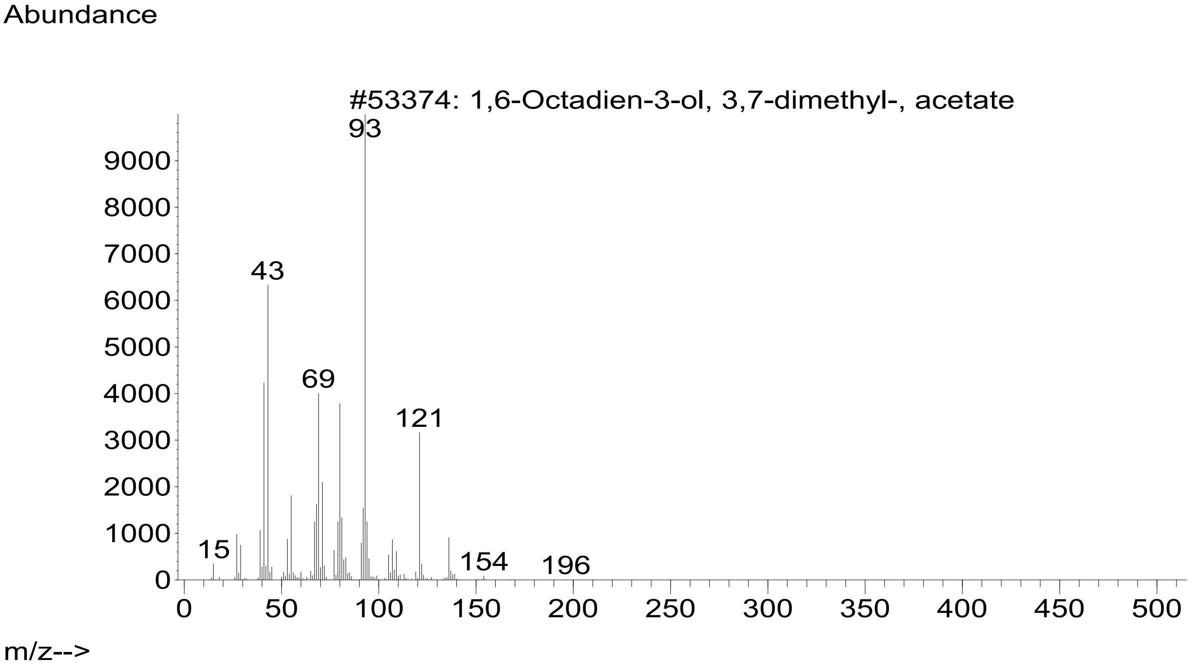

Supplementary Figure S4. Mass spectrum of 4-terpineol present in *Lavandula angustifolia* essential oil, compared with 4-terpineol standard mass spectrum from NIST 02 library

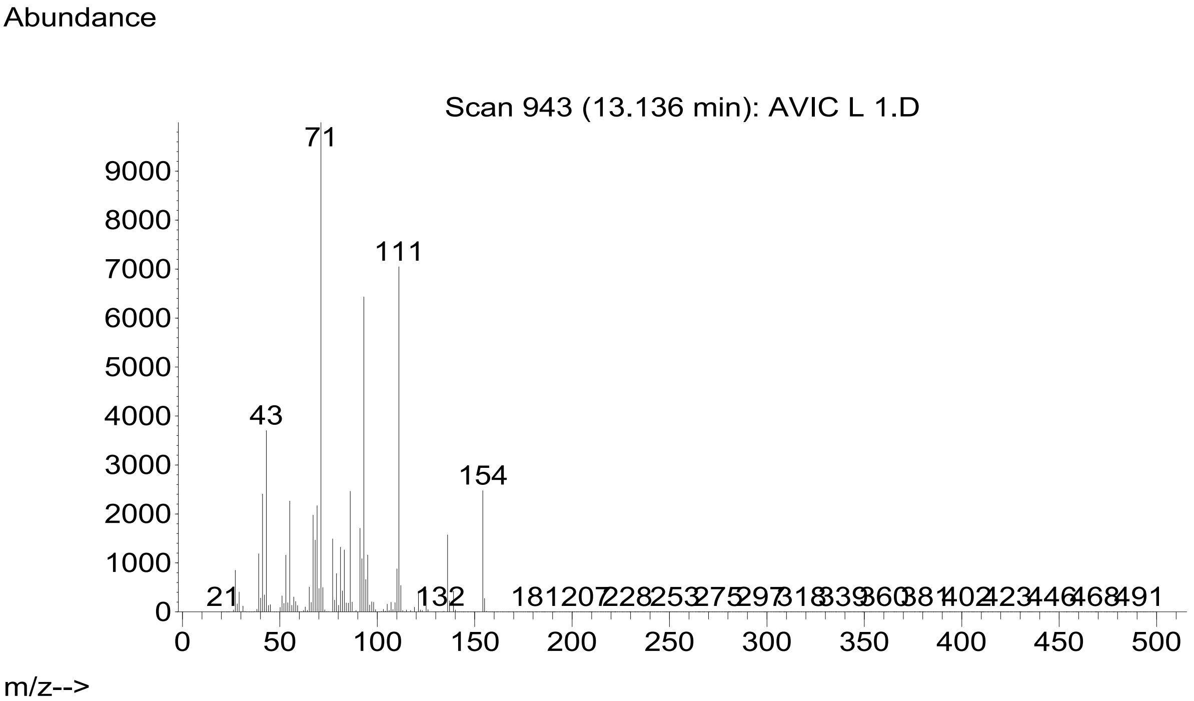

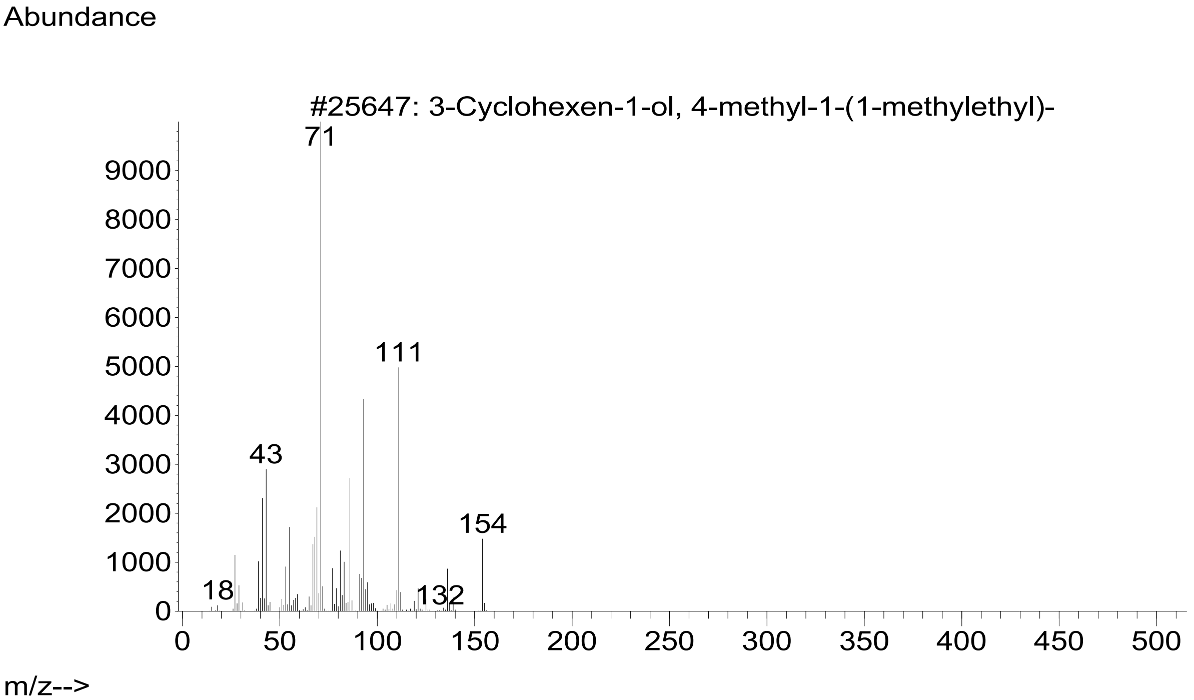

Supplementary Figure S5. Mass spectrum of lavandulyl acetate present in *Lavandula angustifolia* essential oil, compared with lavandulyl acetate standard mass spectrum from NIST 02 library

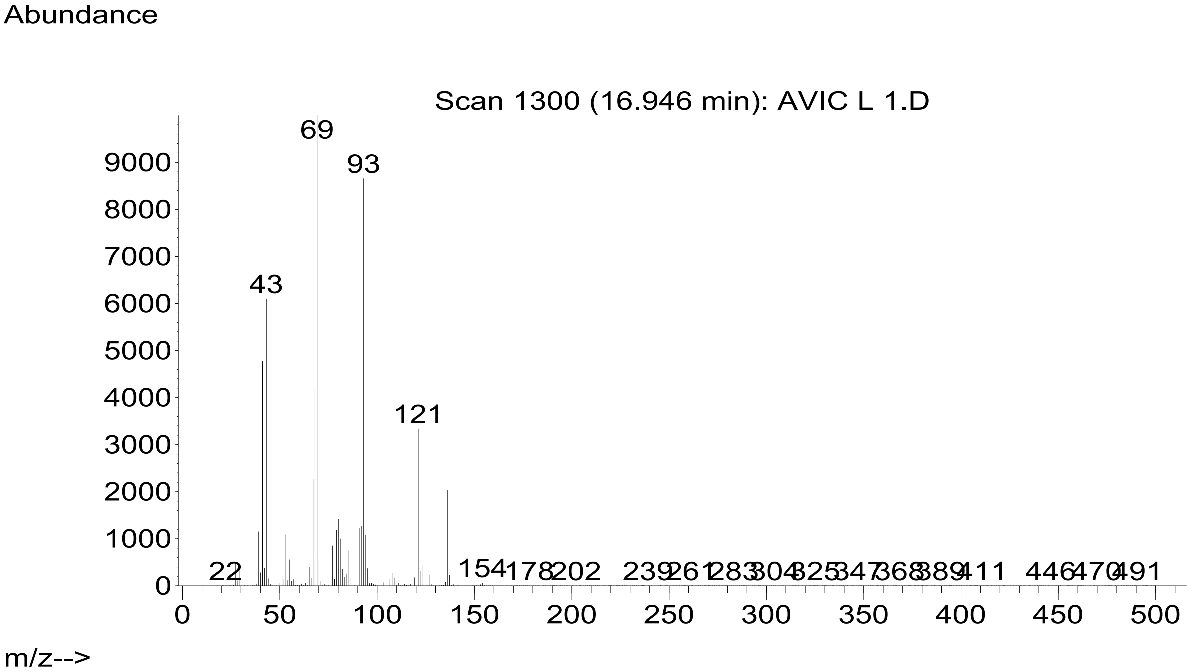

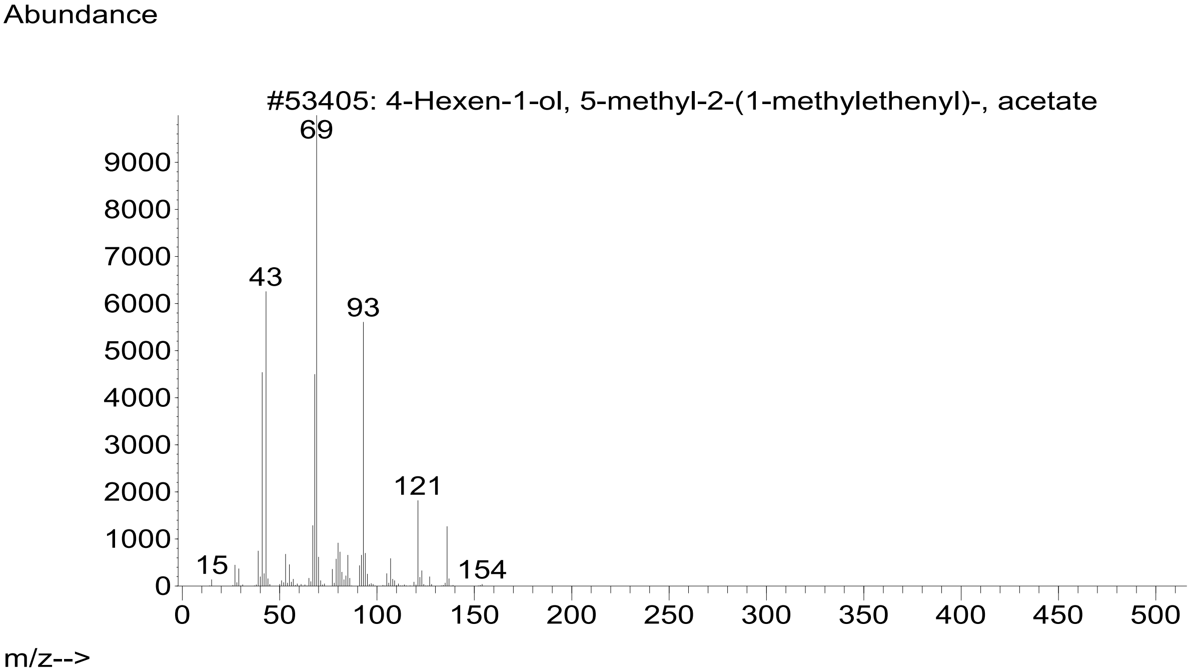

Supplementary Figure S6. Mass spectrum of caryophyllene present in *Lavandula angustifolia* essential oil, compared with caryophyllene standard mass spectrum from NIST 02 library.

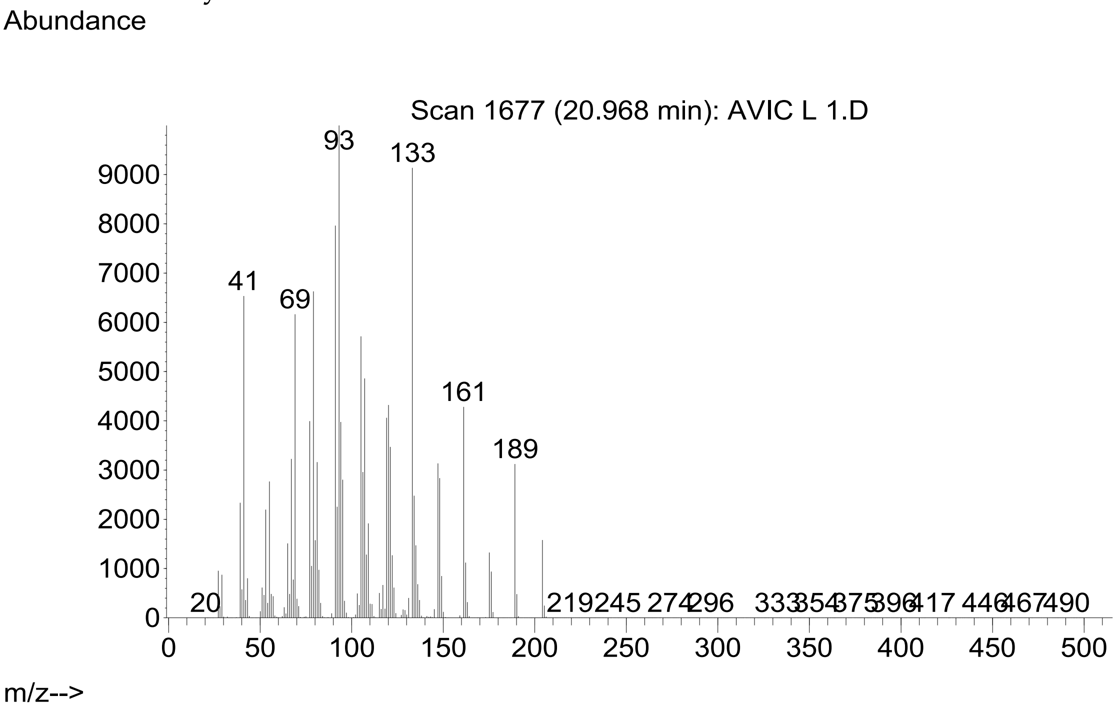

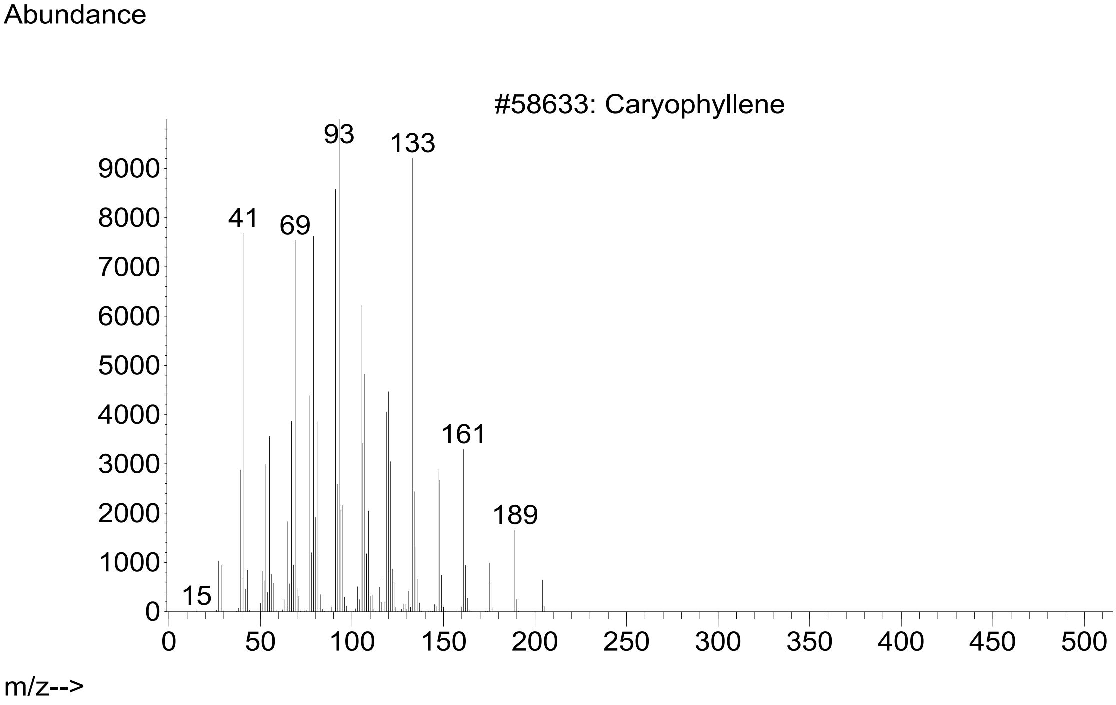

Supplementary Figure S7. Chromatogram of *F.A.M.E. MIX C4-C24* (references material) obtained by GC-MS method

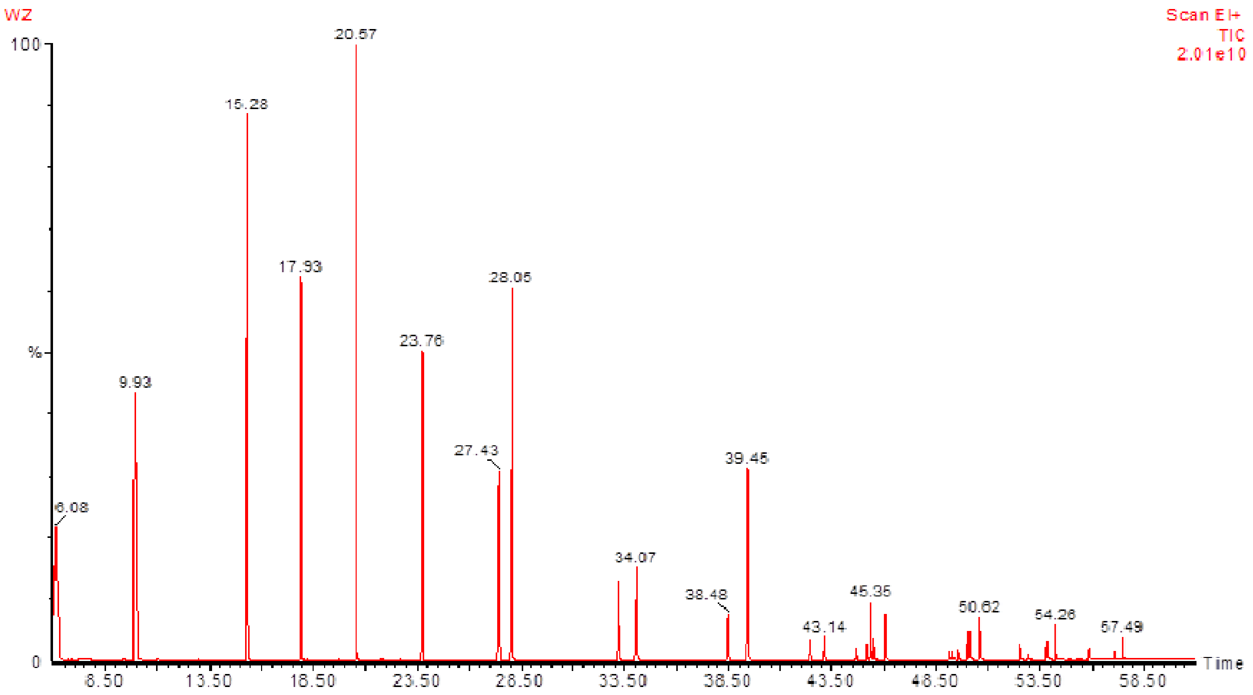

Supplementary Figure S8. Mass spectrum of oleic acid methyl ester (C18:1 n9c) present in breast muscle of broiler chicken, compared with mass spectrum in references material FAMEs mixture

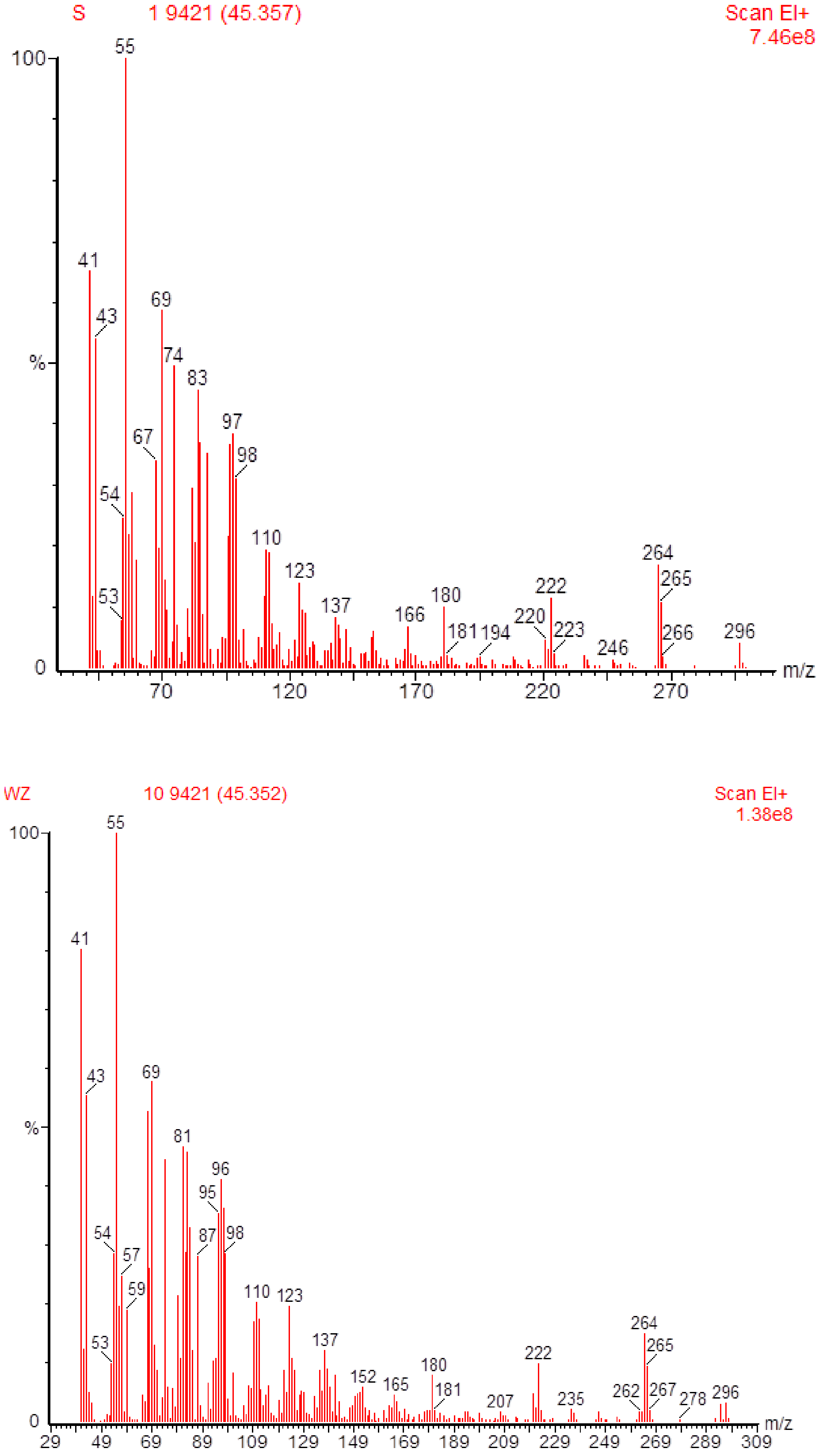

Supplementary Figure S9. Standard mass spectrum of oleic acid methyl ester (C18:1 n9c) from NIST 02 library.

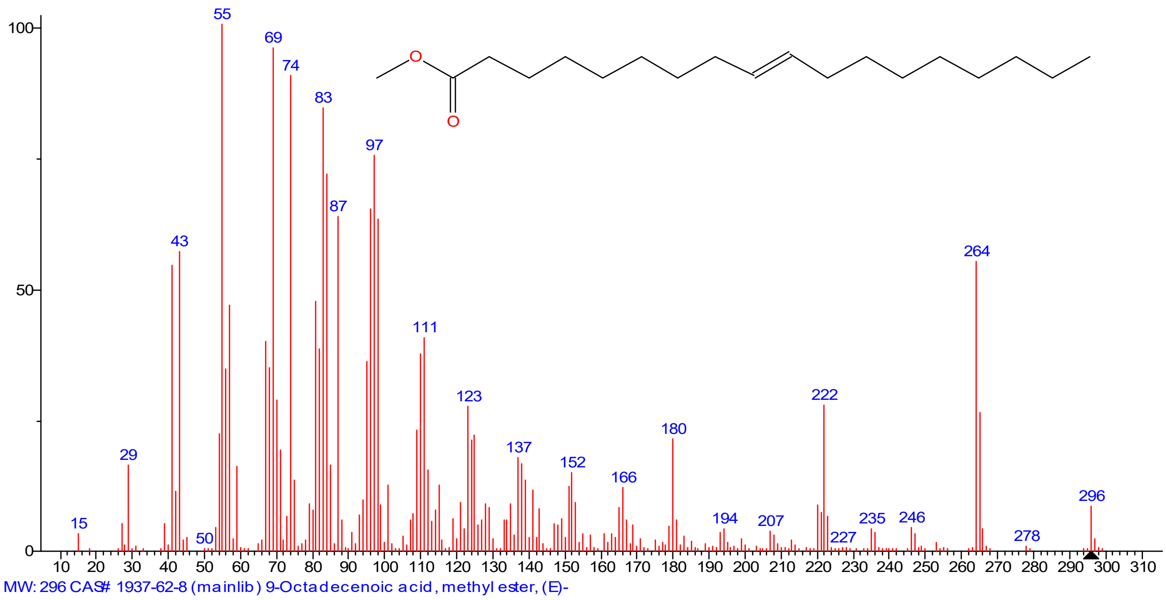

Supplementary Figure S10. Mass spectrum of elaidic acid methyl ester (C18:1 n9t) present in breast muscle of broiler chicken, compared with mass spectrum in references material FAMEs mixture

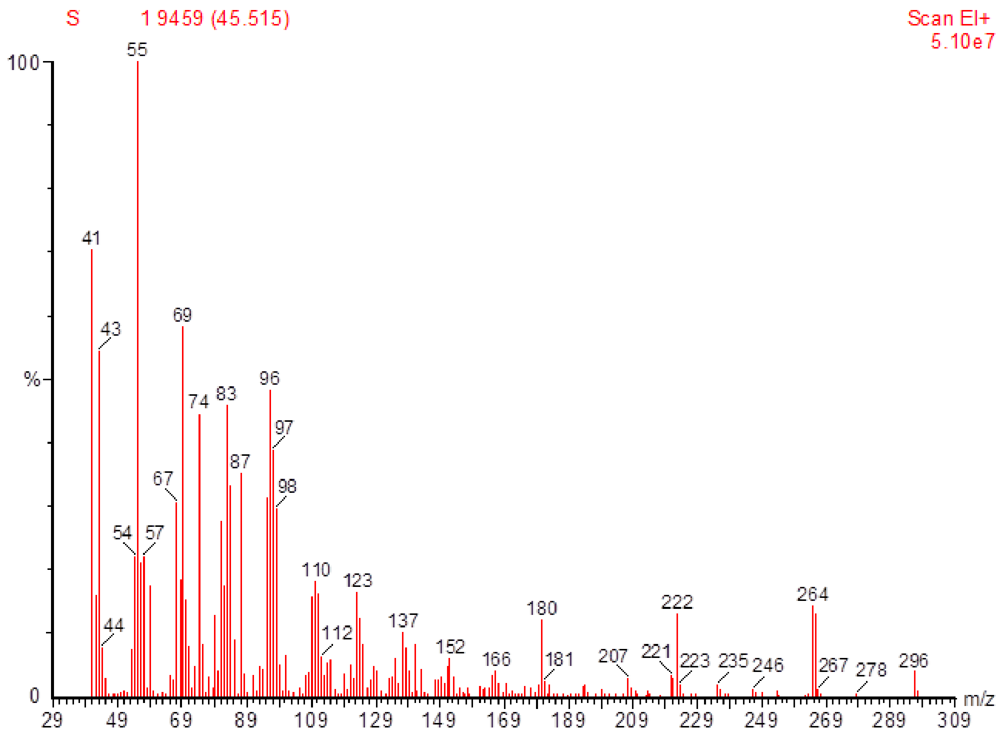

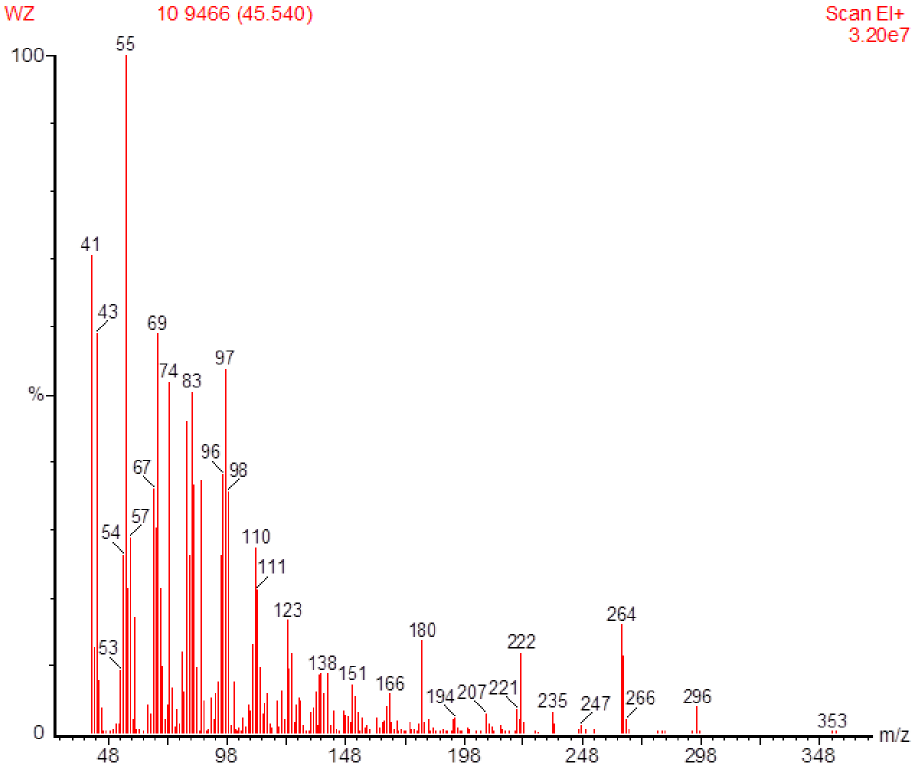

Supplementary Figure S11. Standard mass spectrum of elaidic acid methyl ester (C18:1 n9t) from NIST 02 library.

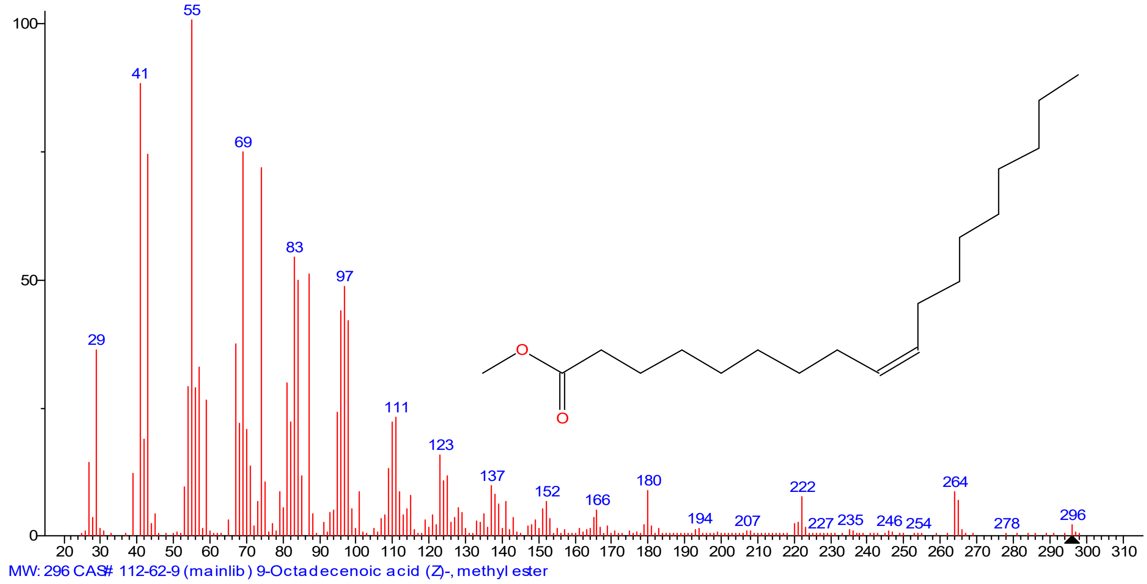

Supplementary Figure S12. Mass spectrum of palmitic acid methyl ester (C16:0) present in breast muscle of broiler chicken, compared with mass spectrum in references material FAMEs mixture

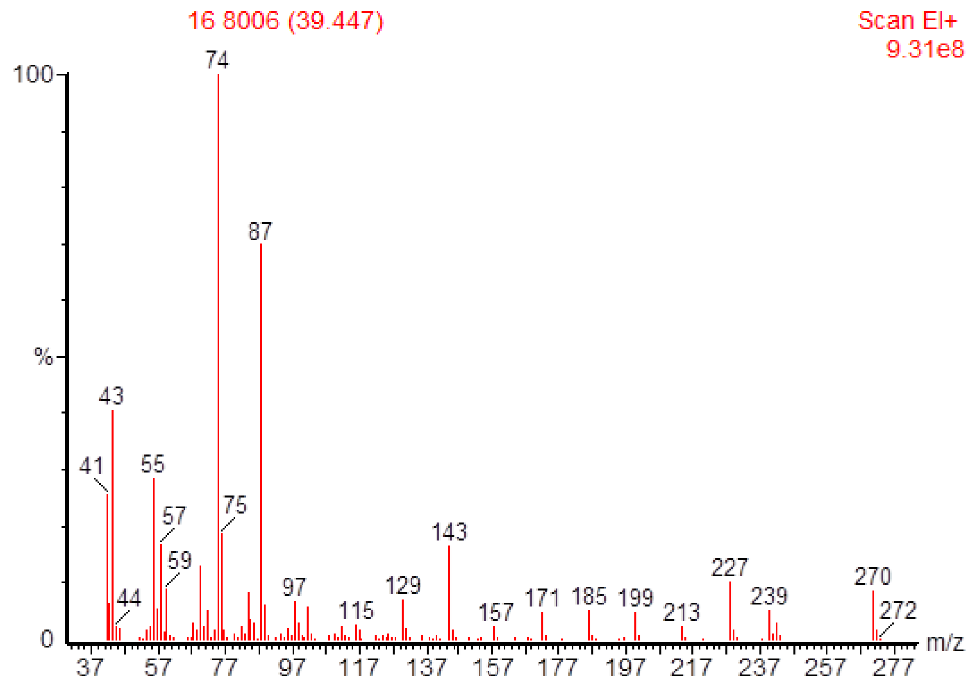

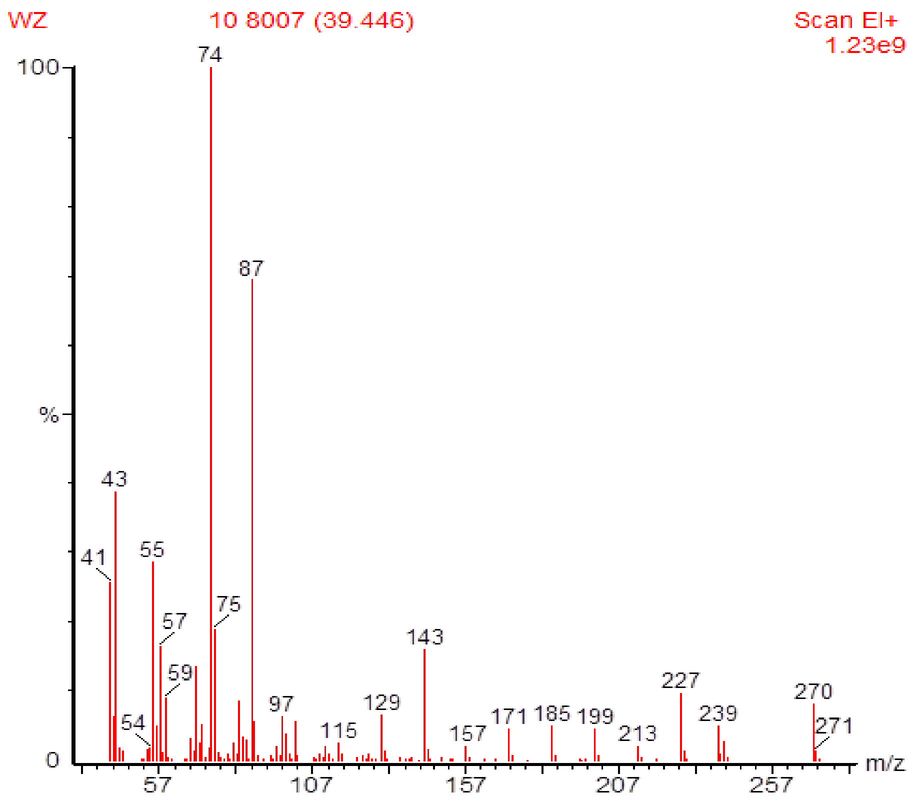

Supplementary Figure S13. Standard mass spectrum of palmitic acid methyl ester (C16:0) from NIST 02 library.

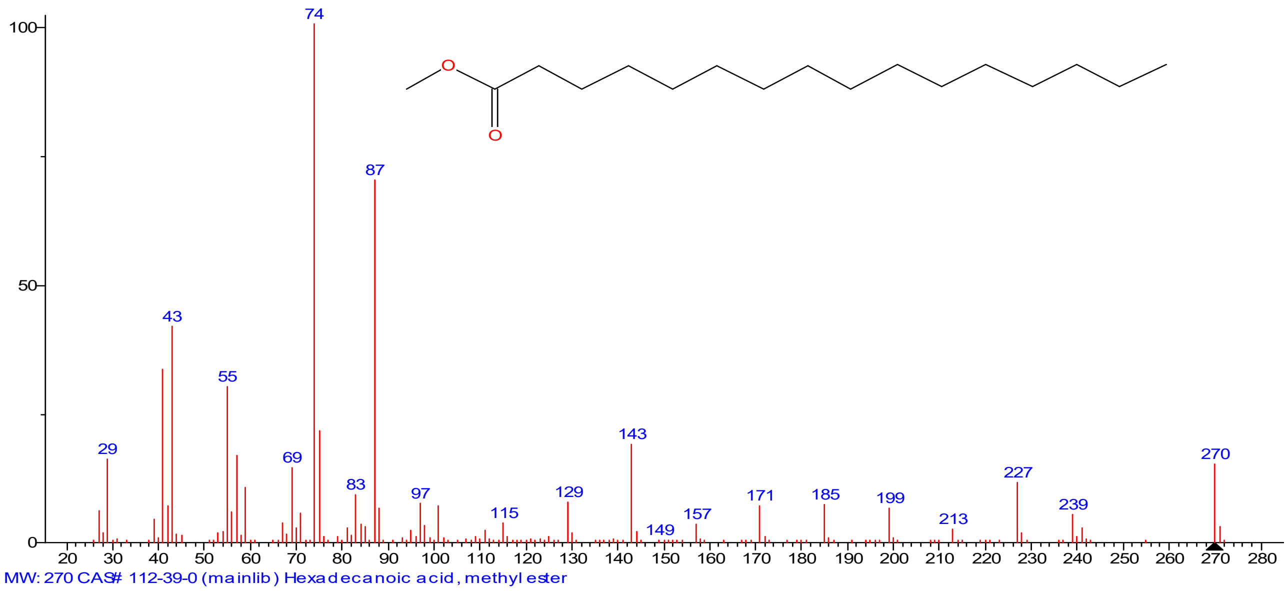

Supplementary Figure S14. Mass spectrum of palmitic acid methyl ester (C16:0)

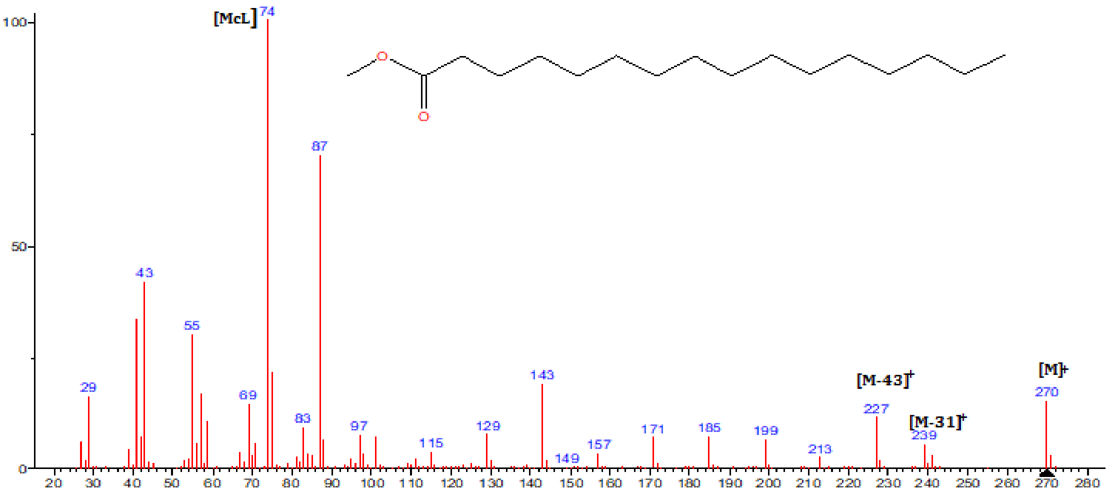

Supplement: Supplementary file 1 [file foods-14-03409-s001.zip › foods-3908062-supplementary.pdf]
